# Monitoring and Behavior of Biomotor Skills in Futsal Athletes During a Season

**DOI:** 10.3389/fpsyg.2021.661262

**Published:** 2021-05-28

**Authors:** Ricardo Stochi de Oliveira, João Paulo Borin

**Affiliations:** ^1^Max Planck University Center (UNIMAX) - Sports and Physical Education Department, Indaiatuba, Brazil; ^2^Sports Training Theory and Methodology Group (GTMTD), Campinas, Brazil; ^3^University of Campinas (UNICAMP) - Physical Education College, Campinas, Brazil

**Keywords:** training load, futsal, monitoring, physical performance, training

## Abstract

Futsal is a sport that presents alternation of high and low intensity moments, which lacks investigations regarding the effects of the organization of the training load on biomotor skills. In this sense, this study aims to verify the monitoring of the training load throughout the season and the behavior of biomotor skills in futsal athletes. Twelve futsal athletes (24.5 ± 4.9 years, 1.79 ± 0.6 m, 72.4 ± 9.4 kg, and 9.4 ± 4.3% fat) from the adult category who competed in the first division of the Paulista championship participated in the study. Throughout the season the internal training load (ITL) was calculated, through the relationship between volume (minutes) and the rate of perceived exertion (RPE), monotony, and training strain. The training periods were divided into: preparatory, competitive and competitive II, for a total of four moments of evaluation: M1: at the beginning of the preparatory period; M2: 5th week, at the end of the preparatory period; M3: 13th week, in the middle of the competitive period; and M4: at the start of the competitive period II. The tests used were: (i) Power of lower limbs: counter movement jump (CMJ); (ii) Displacement speed, over the 10-meter distance (V10m); and (iii) Aerobic power, by the Carminatti test (T-CAR). The variables analyzed were compared at the different moments of evaluation, normally distributed variables (Volume, S-RPE, strain, and monotony) were analyzed using the ANOVA ONE-WAY variance test followed by the Tukey. Variables that did not show normality (lower limb power, speed, and aerobic power) were compared using the Friedman test followed by Dunn's multiple comparisons test and was presented by median and interquartile interval. The significance value adopted was *p* < 0.05. A significant improvement (*p* < 0.05) was observed in the power of lower limbs from M1 (37.5 ± 5.5 cm) to M3 (40.8 ± 5.7 cm), from M2 (38.9 ± 5.5 cm) to M3 (40.8 ± 5.7 cm), and from M1 (37.5 ± 5.5 cm) to M4 (40.2 ± 5.4 cm). Aerobic power showed a significant increase (*p* < 0.05) from M1 (12.1 ± 0.7 km/h) to M3 (12.7 ± 7 km/h) and from M1 (12.1 ± 0.7 km/h) to M4 (12.73 ± 1.04 km/h). The internal training load showed a difference between competitive I and II in relation to the preparatory period (*p* < 0.05). In conclusion, the proposed training organization was sufficient to improve the power of the lower limbs and the aerobic power.

## Introduction

Futsal started in the 1930s in South America as an indoor version of football and has since expanded rapidly around the world. Currently, the sport has its rules governed by FIFA and is practiced by more than 130 countries. The world cup takes place every 4 years, and since 2012, 24 teams have taken part in the competition. In recent years, numerous changes regarding the rules have been made, making the sport more dynamic and intense (Matzenbacher et al., 2014).

The popularity of the sport has fostered research in different domains: physical (Barbero-Alvarez et al., 2008, 2015; Alvarez et al., 2009; Castagna et al., 2009; Milanez et al., 2011; De Oliveira Bueno et al., 2014; Naser et al., 2017; Ayarra et al., 2018; Yiannaki et al., 2020), physiological (Barbero-Alvarez et al., 2008; Oliveira et al., 2013; Arruda et al., 2015; Wilke et al., 2016; Padoin et al., 2020), and control of the training load (Miloski et al., 2014, 2016; Beato et al., 2017; Clemente et al., 2019).

Those previously cited bodies of research characterized futsal as an intermittent modality, alternating high-intensity efforts with short periods of recovery, requiring energy supply both from the aerobic pathway considered predominant during the match and from the anaerobic pathway, the latter being determinant, since actions such as sprints, changes of direction, accelerations, and decelerations can be decisive for the success in the match (Beato et al., 2016). In a study by Ribeiro et al. (2020), it is shown that one of the important loads imposed on athletes is the deceleration that happens during changes in direction and technical and tactical actions. This finding corroborates the studies on the development and improvement of neuromuscular abilities for optimal performance in futsal matches that were mentioned above.

Based on the characteristics of the actions of the sport, there is a pattern in the prescription of training means and methods, both directed to tasks that develop endurance capabilities as well as to the training of the neuromuscular capabilities of athletes to obtain a better competitive performance; however, this seems to be a challenge for coaches and physical trainers due to the concurrent effect that exists in futsal (Nakamura et al., 2016a). An effective strategy to overcome this problem and understand the dynamics of the training load distribution, is focusing on monitoring the training load imposed on the athlete. In team sports, the training load ca be divided into external training load which is the training prescribed and, internal training load which is the training effect (Issurin, 2019).

The ultimate goal of training is to obtain positive adaptations through appropriate training loads. For this to occur, it is necessary to correctly manipulate training variables such as volume and intensity, as well as offering adequate recovery periods. A path to monitoring the training load is and session-RPE designed by Foster (1998). In team sports, especially in futsal, s-RPE is widely used because of its applicability, ratability and internal consistency (Haddad et al., 2017). The session-RPE method takes into consideration the duration of the training session, expressed in minutes and the intensity is given by an athlete through the RPE scoring the intensity of the training session. Thus, the training load is the product of the volume and intensity.

Classical studies on futsal investigated training load. Miloski et al. (2012), who sought to understand the quantification of internal and external load in a 14-week futsal season, presented training parameters. For instance, the ITL was quantified using the S-RPE method (Foster, 1998) and found that the ITL was higher during preseason than during the competitive period, but they also recognized one limitation of the study, which was the need for further investigation into the performance of athletes along with the preparatory and competitive periods. Another study led by Oliveira et al. (2013) shows interesting results regarding physical changes during a training macrocycle (preseason and season) and changes in heart rate variability. Miloski et al. (2016) quantified the ITL, using S-RPE, during 22 weeks in a futsal team. The authors classified the ITL in four different zones, such as being ITL ≤ 25% (of maximum): low loads; 25–50%: moderate-low loads; 50–75%: moderate-high loads; and ≥75%: high loads; however, no data were investigated regarding the distribution of volume and intensity, as well as the relationship with training organizations and biomotor capabilities.

Other studies aimed to investigate the organization of training and its response either on physical performance or results in a competition. For instance, Thiengo et al. (2013) investigated the organization of training loads on biomotor capabilities quantifying the volume of training in 5 weeks of the preparatory period for amateur futsal players. As a result, the authors found that 1,530 min of training is subdivided into 40% of metabolic and technical/tactical training, and 60% on neuromuscular training, such as strength, power, and speed. In response to this form of organization, the athletes achieved 13% of improvement in maximum anaerobic power and 4.4% of improvement in the power of lower limbs. Cetolin and Foza (2010) studied under-20 futsal players for 4 months and monitored the athletes' external load during the whole season. For the first month, the training emphasis was based on specific endurance and aerobic power; for the second month, the activities focused on strength and speed; lastly, in the third and fourth months, speed training was increased. As a result, the team won more than 70% of the matches during the competition.

Rocha et al. (2015) studied the changes in performance and biochemical indicators of a team. In addition, Teixeira et al. (2018) monitored and compared the quantification of the training load in two futsal teams and observed the metabolic and neuromuscular responses; however, that investigation, although necessary, occurred only in the first 5 weeks that characterized the preparatory period. Lago-Fuentes et al. (2020) described both external training load and ITL in a professional female futsal team during 43 weeks and compared workloads during different periods of the season. The authors found interesting results relating to the pattern of workload organization throughout the season. Wilke et al. (2021) compared the post-training recovery timeline of elite Brazilian futsal players during 10 weeks of preseason. In summary, they found that the organization of training attenuated the perception of effort and fatigue of players, therefore improving the recovery of power, muscle damage, and vigor markers.

To the best of our knowledge, most studies regarding futsal are specific to one period of the season, investigating hormonal responses, quantifying the intensity of training, and evaluating the changes in performance. Albeit interesting, it appears to be a limiting factor. From the aforementioned research, few studies investigated the entire training season, and none quantified the relationship between volume and intensity in biomotor capabilities responses. For instance, all the studies investigated the training load proposed by Foster (1998), but none of them indicated whether the training load was greater because of the training volume or intensity at different periods of the season. Given the above, there is a hypothesis that either the volume or intensity of training may affect the total training load. Furthermore, the dynamics of these variables can cause positive or negative effects on athletes' body.

Thus, the present investigation is justified for it contemplates research within a complete season, encompassing both preparatory and competitive periods. The objective, therefore, is to verify the monitoring of the training effect and to compare the responses of biomotor skills at different times of the competitive season.

## Materials and Methods

### Participants

Twelve futsal athletes who competed in the first division of the Paulista Championship participated in this study (24.5 ± 4.9 years, 1.7 ± 0.6 m, 72.4 ± 9.4 kg, and 9.4 ± 4.3% fat). All the athletes had at least 5 years of experience in the sport, having played in the national and international championships. Participants were informed about the study by the responsible researcher and subsequently signed the informed consent form that was previously approved by the Research Ethics Committee of the State University of Campinas, under CAAE no. 48065615.0.0000.5404.

### Design and Procedures

**Figure 1:**
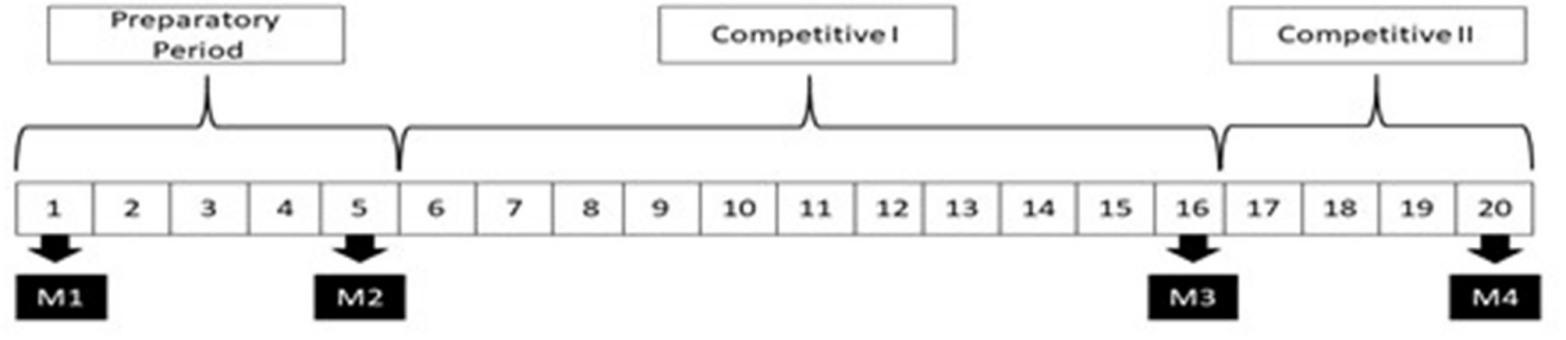
Periods of training and assessment.

The training macrocycle consisted of 20 weeks, 5 in the preparatory period, 11 in the competitive period I (qualifying phase), which featured 12 official games, and 4 weeks of competitive period II (play-offs). Figure 1 illustrates the experimental design of the study along with the moments of the evaluations (M). Tests were carried out in order to assess aerobic power, power of lower limbs, and speed. To avoid any kind of influence on the results and as a form of standardization, athletes performed a battery of tests with, at least, 48 h of rest. The speed and power tests of the lower limbs were performed in the morning between 9:00 a.m. and 12:00 p.m., and the aerobic power test was performed in the afternoon between 3:00 p.m. and 5:00 p.m. The athletes wore their own training clothes, such as t-shirts, shorts, socks, and indoor soccer shoes. The tests were applied by the responsible researcher and the technical staff.

### Training Program

The training schedules during the season were planned entirely by the coaching staff of the team without intervention by the authors of this study. The team aimed to develop technical tactical skills, strength-power training, aerobic power, and, finally, matches (Table 1). The athletes trained once or twice a week, depending on the schedule of the coaching staff of the team. The training week consisted of 7 days containing a minimum of 7 training sessions and a maximum of 11 training sessions. The preparatory period had a range of 9–11 sessions per week, and competitive I and II had a range of 7–9 sessions per week. Each session lasted an average of 90 min. After each training session, one of the authors of the study gathered with the technical staff to compute the training sessions and then quantify according to the classification in Table 1.

**Table 1:** Training content.

**Training**	**Content**
Technical–tactical	• Transition games (attack–defense, defense–attack) with and without a numerical difference; simulated games training
Strength–power	• Strength training for lower and upper limbs: 3 to 4 sets of 8 to 15 maximum repetitions, with 2 min of recovery between sets and 3 min between exercises • Strength training for lower and upper limbs: 3 to 5 sets of 2 to 5 maximum repetitions, with 3 to 4 min of recovery between sets and 5 min between exercises • Resisted runs 10 to 15 m • Vertical jumps with heights of 20 to 70 cm, horizontal jumps and unilateral jumps • Plyometric training using different drop heights (20, 40, and 60 cm).
Endurance and specific endurance training	• Extensive interval runs with stimuli of 8 to 10 min at threshold speed with intervals of 3 min of recovery • Intensive interval runs, with 4-min stimuli at 90% of maximum heart rate, and with 3-min recovery at 70% of maximum heart rate • Small sided games (1 × 1, 2 × 2, 3 × 3, 4 × 4)
Matches	• Training games and official matches

### Monitoring of External Load

The strength and conditioning coach of the team along with one of the authors recorded day-to-day training in digital diaries designed by technical staff (Excel spreadsheet). The training was recorded for each session and included total training time distributed across training form (endurance, specific endurance, strength, power, technical/tactical drills, and matches). In each week, the total weekly external training load was calculated by adding the training minutes, and the total external load (training time) of each period was estimated as the mean of the total weekly training load.

### Monitoring of Internal Load

To calculate the training load according to the procedures described by Foster et al. (2001), the training time (in minutes) of each session was measured, as well as the intensity, through the S-RPE reported by the athletes. The scale is graded from 0 to 10 points, with the value 0 (zero) representing no effort and 10 (ten) representing the maximum perceived effort. S-RPE was recorded 30 min after each training session, after the reported RPE score was multiplied by total session duration, in minutes, to indicate the training load. In each week, consisting of 7 days, the total weekly training load was calculated by adding the training loads of the session. The total ITL of each period was estimated as the mean of the total weekly training load. During the competitive phase, the RPE of the match was also recorded. The time that each athlete was on the court was added and multiplied by the reported RPE value. The monotony and strain were also calculated. Monotony indicates the load variability between training sessions and was obtained by the ratio between the mean and the SD of the internal load. The strain is associated with high monotony and might indicate overtraining and was obtained by multiplying the total training load by monotony. The mean of the total training load, strain, monotony, RPE, and training volume was calculated for better visualization of the external and internal loads (Foster, 1998; Foster et al., 2001).

### Testing Protocol

#### Power of Lower Limbs

To evaluate the power of the lower limbs, the vertical jump test with the countermovement jump technique (CMJ) was used according to the protocol proposed by Bosco et al. (1983). Each athlete made three attempts with a 10-s interval, the highest value among them was used. To perform the test, a CEFISE® contact mat connected to a portable computer was used, and from the time of flight, the height of the jump was calculated using specific software.

#### Displacement Speed

The protocol proposed by Little and Williams (2005) was used to evaluate the displacement speed over 10 m. The athlete positioned himself, standing in the starting line, and at the command of the evaluator, ran at maximum effort in order to cover the stipulated distance in the shortest possible time. Three attempts were made, with an interval of 2 min between them, the best of which was computed. Two CEFISE® photocells were used for the evaluation of the displacement speed, being placed at the start and end points of the test.

#### Aerobic Power

To measure the aerobic power of the athletes, the incremental intermittent running test, T-Car, (Da Silva et al., 2011) was used. The test consisted of 90-s stages. In each stage, 5 repetitions of 12 s of running were performed (6 s “to go” and 6 s “to come back”) interpolated with 6 s of recovery. The pace was dictated by an audio signal (beep), with regular intervals of 6 s that determine the running speed in the displacements predicted in each stage.

### Statistical Analysis

The data obtained were tabulated and analyzed using GraphPad Prism 8.0 for Windows. After analyzing the data distribution using the Shapiro–Wilke test, normally distributed variables (volume, S-RPE, strain, and monotony) were analyzed using the one-way ANOVA test followed by Tukey's range test. Variables that did not show normality (lower limb power, speed, and aerobic power) were compared using the Friedman test followed by Dunn's multiple comparisons test and were presented by the median and interquartile interval. The magnitude of effect for pairwise comparisons was analyzed using Cohen's d with a 95% confidence interval. The magnitude of d was qualitatively interpreted using the following thresholds: <0.2, trivial; 0.2 to 0.6, small; 0.6 to 1.2, moderate; 1.2 to 2.0, large; and 2.0 to 4.0, very large (Cohen, 1994).

## Results

**Figure 2:**
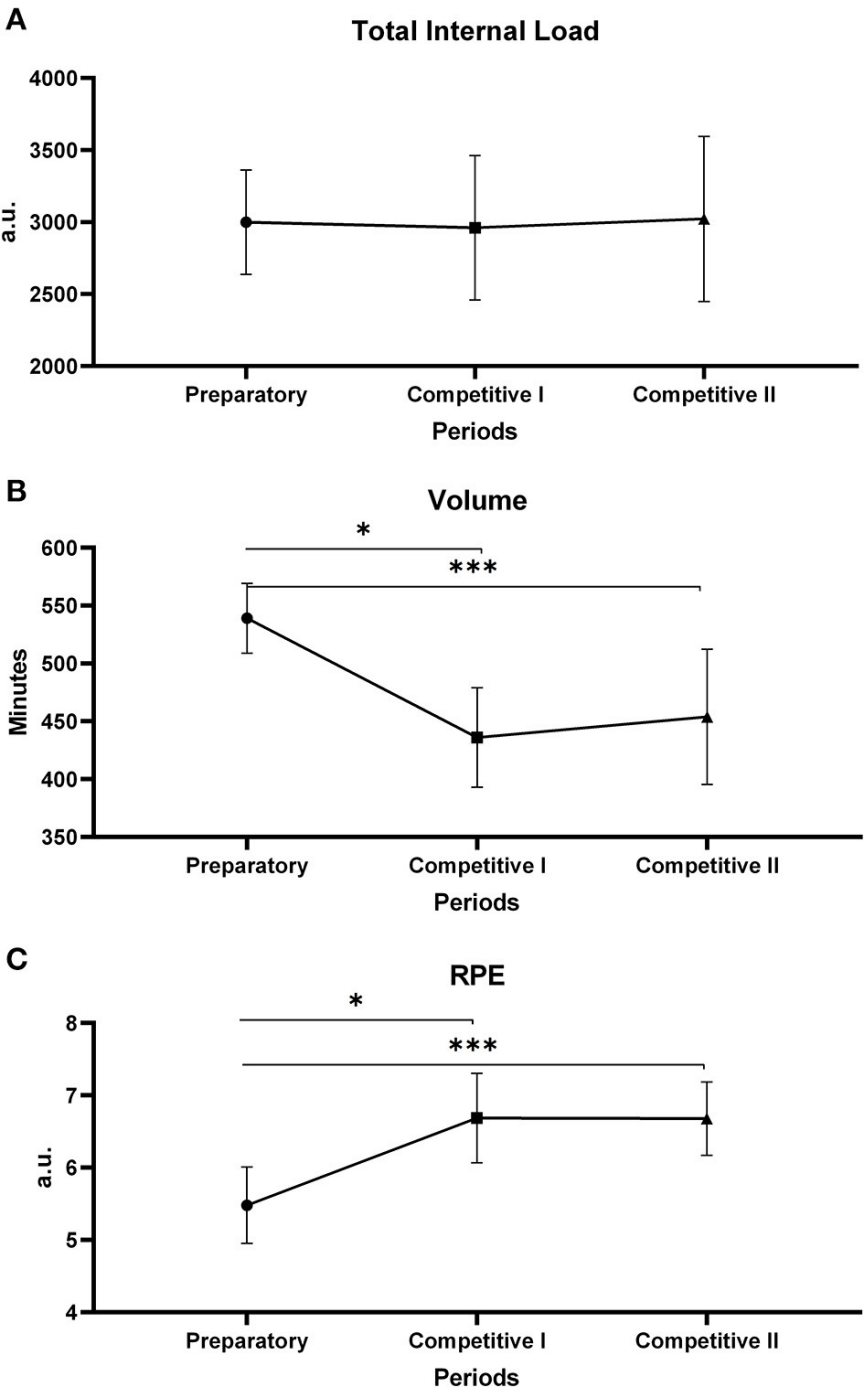
**(A)** The total internal training load between periods. **(B)** Training volume between periods. **(C)** S-RPE between periods. *Difference between preparatory period and competitive period I, ***Difference between preparatory period and competitive period I.

**Figure 3:**
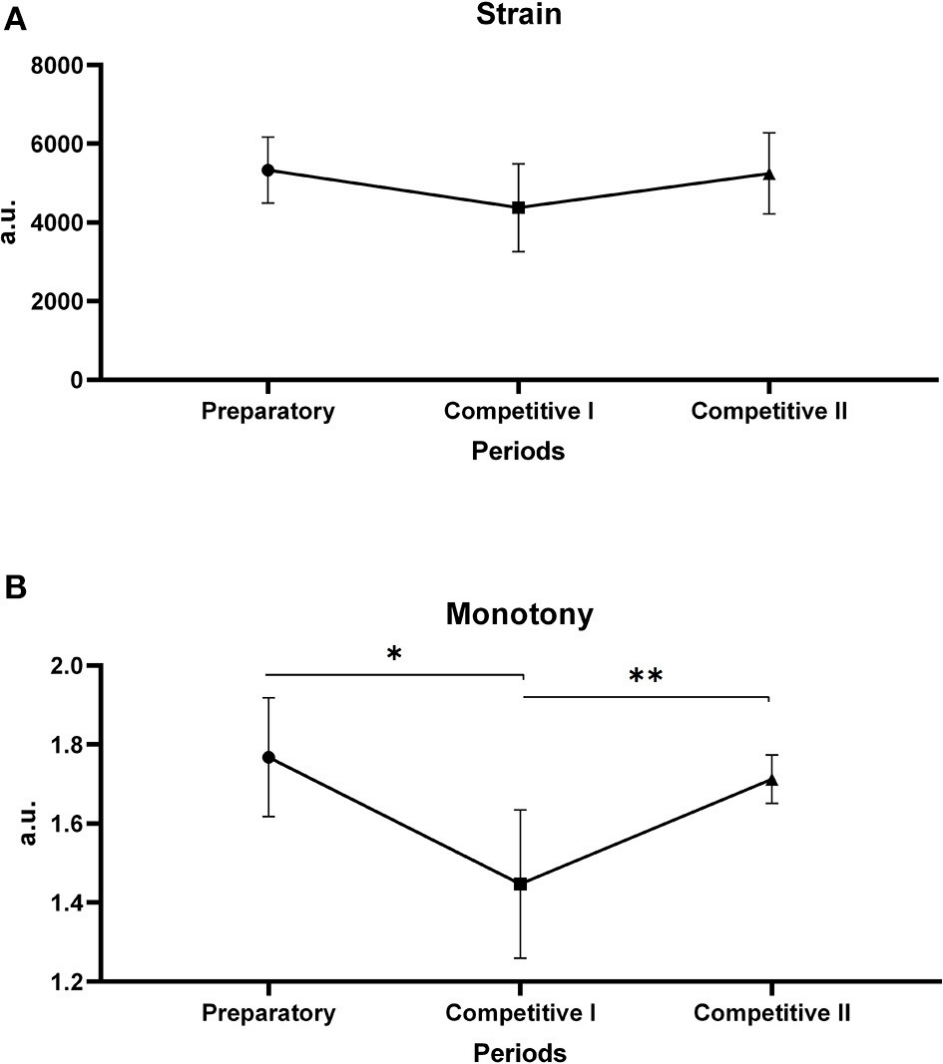
**(A)** The strain between periods. **(B)** Monotony between periods. *Difference between preparatory period and competitive period I, **Difference between competitive period and competitive period II.

The results are presented below by training periods at mean values and SD. Figure 2 points to the distribution of the total ITL (A), the volume (B), and the RPE of the session (C). Regarding volume, the preparatory period was higher by 539.0 (30.2) min than other periods (*p* < 0.01), with no significant differences between competitive period I 435.9 (42.8) and II 453.8 (58.5). The S-RPE presented higher values (*p* < 0.03) between competitive I 6.7 (0.61) a.u. and II 6.8 (0.50) in relation to the preparatory period 5.3 (0.5) a.u. Figure 3 shows monotony and strain, in which the monotony shows a significant difference (*p* < 0.01), revealing that the preparatory 1.7 (0.15) a.u. and competitive II 1.7 (0.06) a.u. periods presented higher values than competitive I 1.4 (0.8) a.u. The strain showed no difference between the periods.

**Figure 4:**
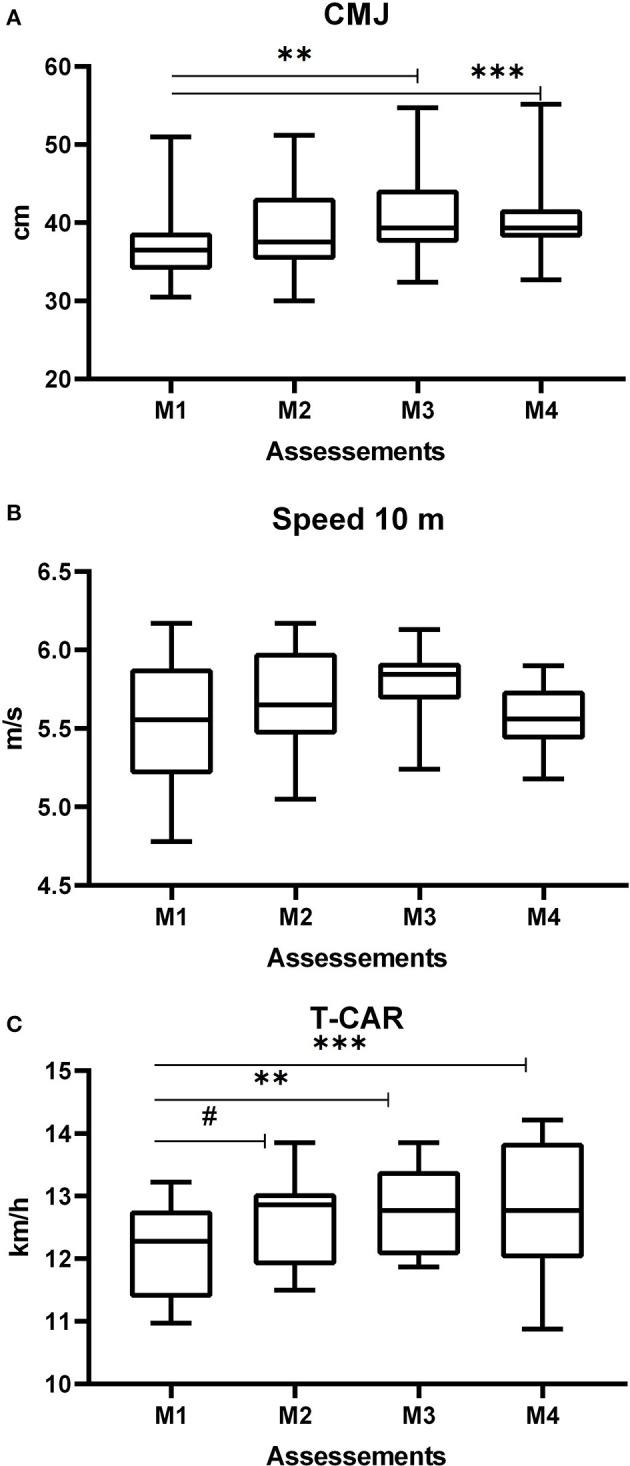
**(A)** CMJ. **(B)** Speed 10 meters. **(C)** T-CAR. ^#^Difference between M1 and M2, **Difference between M1 and M3, ***Difference between M1 and M4.

**Figure 5:**
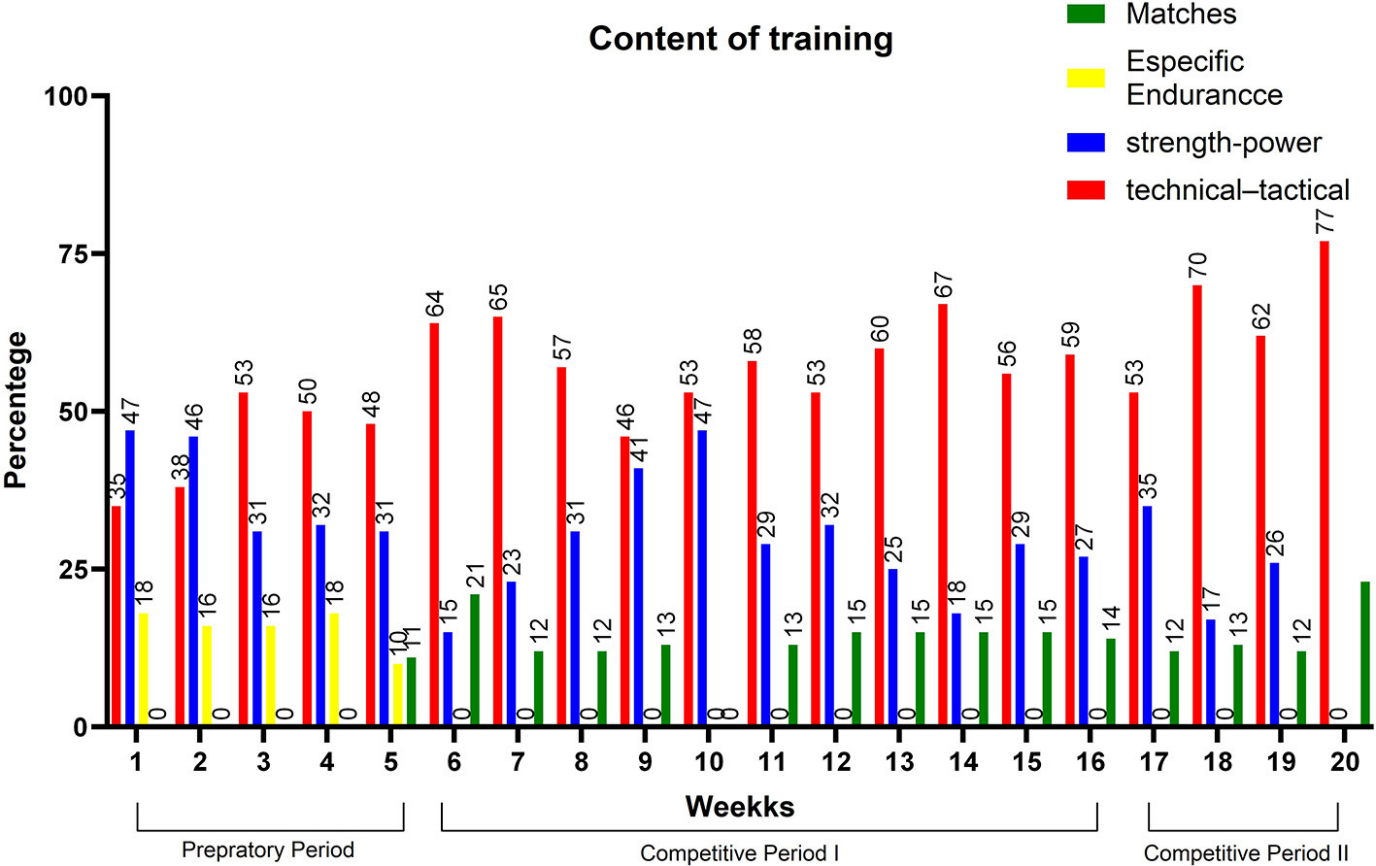
Relative training volume.

Figure 4 and Table 2 present the results of the biomotor capabilities (CMJ, Speed, T-CAR). CMJ improved from M1 [37.5 (5.5) cm] to M4 [40.2 (5.4) cm] and from M1 [37.5 (5.5) cm] to M3 [40.8 (5.7) cm (*P* < 0.05)]. T-CAR shows a significant difference (*p* < 0.05) between M1 [12.1 (0.7) km/h] and M2 [12.8 (0.6) km/h], between M1 [12.1 (0.7) km/h] and M3 [12.9 (0.7) km/h], and lastly between M1 [12.1 (0.7) km/h] and M4 [12.7 (1.0) km/h]. Speed tests presented moderate changes between M1 and M3 (d = 0.68; moderate). Figure 5 shows the time allocated for different training content, in percentage values. It is immediately possible to observe throughout the macrocycle the predominance of technical–tactical training, except in weeks 1 and 2.

**Table 2:** Effect size and descriptive measures of biomotor capabilities at different times using Cohen's d with 95% confidence interval.

**Descriptive measures**		**M1**	**M2**	**M3**	**M4**	**Effect size**	**M1-M2**	**M1-M3**	**M1-M4**	**M2-M3**	**M2-M4**	**M3-M4**
CMJ	Mean	37.5	38.9	40.8^*^	40.2^**^	ES	0.26	0.59	0.51	0.34	0.25	0.58
cm	SD	5.5	5.5	5.7	5.4	Cohen	Small	Moderate	Moderate	Small	Small	Moderate
Speed 10 m	Mean	5.5	5.6	5.7	5.5	ES	0.4	0.67	0.13	0.26	0.43	0.83
m/s	SD	0.4	0.3	0.2	0.2	Cohen	Small	Moderate	Small	Small	Moderate	Large
T-Car	Mean	12.1	12.6^#^	12.7^*^	12.7^**^	ES	0.63	0.71	0.66	0.12	0.13	0.02
Km/h	SD	0.7	0.7	0.8	1	Cohen	Moderate	Moderate	Moderate	Small	Small	Small

*The magnitude of d was qualitatively interpreted using the following thresholds: <0.2, trivial; 0.2 to 0.6, small; 0.6 to 1.2, moderate; 1.2 to 2.0, large and 2.0 to 4.0, very large*.

# *Difference between M1 and M2*.

* *Difference between M1 and M3*.

** *Difference between M1 and M4*.

## Discussion

The discussion of the data is presented by training periods, so that the preparatory period will be discussed first, followed by competitive periods I and II. The present study sought to verify the monitoring of the training effect and to compare the responses of biomotor capabilities at different times of the competitive season. This study was motivated by the need to understand the organizational structuring of the training load, especially in futsal, since this modality is in the spotlight worldwide and its competitive calendar has a large number of matches. From the data obtained, it is possible to observe that the training organization presented in the study did not show a significant difference in the total training load during the periods analyzed; however, when checking the other variables related to training load, the volume of training was higher in the preparatory period, and the intensity, represented by the S-RPE of the session, was higher in the competitive periods. During the competitive period II, the highest values were found both in the CMJ and in the aerobic power tests, and the lowest monotony values were found in the competitive period I.

It is noted that the training periods are well-defined, even though they have presented different numbers of microcycles between them. As for the preparatory period, it was found that the total ITL does not agree with previous studies that investigated high-level futsal players, such as that of Miloski et al. (2012), and reported higher values of total internal load in the preparatory period. The same pattern of training load distribution was found in another study by Miloski et al. (2016), yet none of them identified which of the variables influenced the training load. In the present study, the volume was predominant in the preparatory period. Such values correspond to the study by Thiengo et al. (2013), where they found high scores in the volume of training in the 5 weeks of the preparatory period in a professional futsal team. Lago-Fuentes et al. (2020) also observed high volumes at the beginning of the season. In fact, these high numbers in the preseason can be explained by the need to develop and improve neuromuscular and metabolic capabilities and tactical technical training.

When looking at Figure 5, it can be seen that, in the first 5 weeks, most of the time was devoted to technical and tactical training, though the strength, power, and endurance capabilities were also developed during this period. This volume distribution made the intensity represented by S-RPE lower in the preparatory period. Moreira et al. (2013) quantified the training intensity in three zones based on the session RPE (low, <4; moderate, >4 au and <7 au; and high, >7 au). Regarding the values mentioned above, we can assume that the S-RPE was considered moderate during the preparatory period. These data are similar to previous studies which were found values between 5 and 6 points on the mean RPE (Teixeira et al., 2018; Lago-Fuentes et al., 2020). So, the present study showed a preparatory period with high volume and moderate intensity. These behaviors can be explained by the number of training sessions during the preparatory period and also by the short time that tam sports, especially futsal, have designated for practices before a competition (Miloski et al., 2012), considering that if both high volume and high intensity were prescribed within the same period, the training load could rise to undesirable levels, causing negative adaptations to the body of the athlete.

It was pointed out by Fessi et al. (2016) that excessive high-intensity training in the preseason is generally associated with greater monotony, strain, fatigue, and muscle pain than during the in-season. Of the indicators mentioned above, monotony was below 2 a.u. (arbitrary units) in the preparatory period. According to Foster (1998) and Suzuki et al. (2006), values above 2 a.u. indicate little variability of the load, causing no adaptation to the training process. Strain is also associated with the level of adaptation to training, in which periods with a high load associated with high monotony can increase the incidence of infectious diseases and injuries. In the present study, the values did not exceed 5,000 a.u., unlike the study of Miloski et al. (2012) with futsal athletes, in which scores lower than those in this study were found at 37 weeks 2,270 ± 1,294 (213 a.u. a 4,771 a.u.). Despite the greater results presented in the study, the values are still within acceptable standards, since according to Foster (1998), values of strain close to or >6,000 a.u. favor the development of diseases and negative adaptations to the body of the athlete.

The organization of the training variables and the response of strain and monotony indicators are reflected in the adaptation to training by the athlete. Such adaptation can be observed in the behavior of biomotor capabilities in the preparatory period. It is worth noting that the evaluations carried out between M1 and M2 coincide with this period and suggest that the preseason did not provide sufficient stimuli for significant gains in neuromuscular capabilities. This can be attributed to the concurrent effect caused by large volumes of training aimed at functional capabilities, such as aerobic resistance and tactical technical training (Loturco et al., 2015). Previous investigations with futsal athletes, such as Soares-Caldeira et al. (2014), found no changes in lower limb power in 4 weeks of preseason. Likewise, Miloski et al. (2016) who investigated 22 weeks of training found no changes in the power of lower limbs in the 6 weeks intended for preparation. The authors attribute this result to the difficulty of promoting the distribution of training in order to develop multiple components (physical, technical, and tactical) during short periods of preparation. Nonetheless, the effect size of the neuromuscular capabilities shows a small effect, while the resistance capability shows a moderate effect improvement.

Although there was no improvement in performance during the preseason due to the short period of time, the fatigue perception and recovery from session to session may have improved. Wilke et al. (2021) compared the post-training recovery timeline of elite Brazilian futsal athletes before and after 10 weeks of the preseason. They presented similar results with this study, showing no improvement regarding CMJ, speed, and endurance capacity. The authors observed that players perceived the session as less intense, which supports the notion that exposure to training may improve the perception of the load and, in turn, perhaps the tolerance to fatigue. Therefore, it was presumed that the ratio between tactical, neuromuscular, and metabolic technical training in the preparatory period triggered positive adaptations in the bodies of athletes, since there was no loss of performance. The time allocated for preseason, however, was too short for significant improvements. Freitas et al. (2012) pointed out that for high-performance futsal, the preparatory period is short and it aims to establish the sport-specific physical preparedness, so that, later on, it can be maintained in the best possible way during the competitive period.

When analyzing the competitive period that consists of the competitive periods I and II, it was clear that the competitive period I was the one with the longest duration and it represented an important part of the competition which was the classificatory stage. In this period, the total training load did not change in relation to the preparatory period; however, it was observed that the training volume decreased significantly from the preparatory period to competitive I, and the S-RPE increased (Figures 2B,C). The mean RPE in our study was 6.7 (0.61) over the competitive period I. These data are higher than previous studies in a season with youth male players (Rabelo et al., 2016), professional male team (Freitas et al., 2014; Miloski et al., 2014), and professional female team (Lago-Fuentes et al., 2020). These dynamics between volume and intensity, on the other hand, is in agreement with the premises found in the classic literature on sports training, which shows that in the preparatory period the training volume is prioritized over the intensity, and in the competitive period there is an inversion, prioritizing the intensity over the volume training (Matveev, 1991, 1997; Forteza de la Rosa, 2006; Gomes and Souza, 2008; Platonov, 2008; Tønnessen et al., 2015).

In addition to the behavior of the volume and intensity variables, the low monotony values in the competitive period I also stand out (Figure 3B). As mentioned above, monotony is a potent indicator of the dynamics of training load prescription, as the adaptation induced by training depends on a few aspects, including the stress-recovery relationship (Borges et al., 2019). Foster (1998) and Suzuki et al. (2006) presented evidence that the good performance of sportspeople, regardless of the sport, is related to low monotony values. In fact, it was observed that the players in the present study were subjected to high intensities in the competitive period I, but with low monotony scores. Iaia and Bangsbo (2010) and Laursen (2010), and Mujika (2010) pointed out that prescribing high training intensities with the correct recovery dose favors positive adaptations in the bodies of the athletes, consequently improving the performance of biomotor capabilities. This premise is reinforced by Teixeira et al. (2018) who analyzed two futsal teams subjected to different training intensities in order to observe changes in physical capabilities and concluded that coaches are advised to oscillate the dynamics of the training load applied between the microcycles and allow for recovery cycles.

When looking at the biomotor capabilities, therefore, it is possible to state that these low monotony values may have caused improvements in the height of the CMJ and, consequently, in the lower limb power levels, mainly from M1 to M3. It is worth mentioning that, in addition to the monotony, the proportion of distribution of neuromuscular training and tactical technical training within this period provided positive adaptations in lower limb power gains. For instance, the present study showed a range of strength training between 15 and 47% (Figure 5) of total training during the competitive period I. These values are higher than that shown in the study of Miloski et al. (2016), which is in the range between 9 and 22.8%, that is dedicated to strength training during a season in professional futsal players. Loturco et al. (2015) observed the little time being devoted to neuromuscular training, which ended up influencing lower limb power gains in soccer players. It is important to highlight that the increase and/or maintenance of power levels is essential for decisive actions in futsal, especially in actions that require changes in direction, acceleration, and deceleration (Ribeiro et al., 2020).

The displacement speed did not improve between the evaluated moments; however, between M1 and M3, the effect size was moderate, indicating a possible positive change in this capability. One reason for this was the distance used, not allowing for changes in the values found. A study by Oliveira et al. (2019) found improvements in the displacement speed in futsal athletes during 18 weeks of training. Another possibility, and perhaps the most plausible, suggests that the result can be partially attributed to the effects of simultaneous training caused by high volumes of both technical–tactical and aerobic training in the preseason and competitive periods. Previous studies (Loturco et al., 2015) with soccer players did not report an increase in neuromuscular capabilities with short preparation periods (4 weeks), and Soares-Caldeira et al. (2014), with futsal players, point to similar results for speed with short-term preparation period. The distribution of training content in the present study may have hindered the speed during the season. It is possible to observe that, from the 13th week forward, the volume destined for neuromuscular capabilities decreased while it increased for technical training.

Regarding aerobic power, there was a significant improvement in the threshold speeds from M1 to and M3 and, mainly, from M1 to M4. Since futsal is a high-intensity intermittent sport with a predominance of aerobic metabolism (Barbero-Alvarez et al., 2008; Castagna et al., 2009), the volume assigned for technical and tactical training, in addition to solving this task, contributed to the improvement and maintenance of aerobic power throughout the season. In the values found in the present study, more than 50% of technical/tactical training are similar to values found by Freitas et al. (2012) and Teixeira et al. (2018). More time allocated to this kind of training can lead the bodies of athletes toward metabolic adaptation. For instance, Wilke et al. (2016) investigated the metabolic demands of technical and tactical training sessions in professional futsal athletes and showed that the athletes exercised at intensities above the respiratory compensation point in 20.4 (11.78)%, between respiratory compensation point and the ventilatory threshold (VT) in 28.2 (5.6)%, and below the ventilatory threshold in 51.4 (9.7)% of the time during training. Based on the data mentioned above, it was observed in the present study that the technical/tactical training sessions may have been intense enough to cause si'sgnificant changes.

In the competitive period II, the load behavior was similar to the CPI, with an increase in monotony values and in the time allocated for technical and tactical training (Figures 4, 5), which may have reflected in the neuromuscular indicators, especially the displacement speed. Teixeira et al. (2018) also observed loss in neuromuscular indicators in futsal athletes, especially in the displacement speed, being attributed to the accumulation of training load without due time for recovery and to the volume of technical–tactical training, especially for faster players, given that in another study (Nakamura et al., 2016b) it was observed that fast players perceive greater training loads and present greater reductions in displacement speed during periods of high training loads. It is possible to notice that the highest volumes of technical–tactical training took place from week 17 to week 20, which agrees with the findings of the studies cited above, showing there was a possible interference of the volume of technical–tactical training along with the increase in monotony on the possible decrease in speed.

## Limitation and Future Directions

A possible limiting factor and a possibility for future studies is the monitoring of training through physiological indicators, such as heart rate, or a biochemical indicator to more accurately understand the responses of a body to the stimuli applied, especially the technical training, since it accounts for a large part of the training throughout the season. Another interesting factor to be explored in future research is the recovery indicators between training sessions, such as questionnaires, physiological indicators, and performance tests after the training sessions. We know that the sample of our study is small, but it is difficult to find professional team sports that would accept being part of research throughout the season.

## Conclusions and Practical Applications

The present study aimed to verify the monitoring of the training effect and to compare the responses of biomotor capabilities at different times of the competitive season. From the collected data, it is possible to conclude that: (i) the proposed training organization was sufficient to improve the lower limb power and the aerobic power of the players and (ii) the dynamics between stimulus and recovery, evidenced by monotony, influenced the performance of the athletes. It is important to highlight the strength of the present study, since there are few studies that analyze training load throughout a season and the behavior of biomotor skills in futsal athletes. In addition, studying sports by high-performance teams within their natural environment is an important step in understanding the responses of all variables involved and providing better advice for coaches.

The production of knowledge having high-performance teams as a source of data has always been an arduous task, especially during a long training period such as the one in this study. In addition, the tools presented here are low cost and easy to use, while also being effective in monitoring training, managing fatigue, and avoiding undesirable adaptations during the season. Coaches and physical trainers must pay attention to the volume of technical and tactical training prescribed so that it does not influence neuromuscular capabilities. This study documented that it is necessary to look beyond the total training load to understand which variable (volume x intensity) should be better dosed; thus, coaches and trainers are advised to look into the variables of training to prescribe exercise to improve performance and apply appropriate workloads during the season and, especially, reducing volume during competitive phases injury risks and decrement of performance.

## Data Availability Statement

The raw data supporting the conclusions of this article will be made available by the authors, without undue reservation.

## Ethics Statement

The studies involving human participants were reviewed and approved by UNICAMP. The patients/participants provided their written informed consent to participate in this study.

## Author Contributions

RS and JB contributed to conception and design of the study. RS organized the database and wrote the first and second draft of the article. JB wrote some sections of manuscript. All authors contributed to manuscript revision, read, and approved the submitted version.

## Conflict of Interest

The authors declare that the research was conducted in the absence of any commercial or financial relationships that could be construed as a potential conflict of interest.
